# In Reply: Does a Screening Trial for Spinal Cord Stimulation in Patients With Chronic Pain of Neuropathic Origin Have Clinical Utility (TRIAL-STIM)? 36-Month Results From a Randomized Controlled Trial

**DOI:** 10.1227/neu.0000000000002922

**Published:** 2024-03-22

**Authors:** Sam Eldabe, Sarah Nevitt, Sara Griffiths, Ashish Gulve, Simon Thomson, Ganesan Baranidharan, Rachel Houten, Morag Brookes, Anu Kansal, Jenny Earle, Jill Bell, Rod S Taylor, Rui V Duarte

**Affiliations:** 1 Department of Pain Medicine, The James Cook University Hospital, Middlesbrough, UK; 2 Centre for Reviews and Dissemination, University of York, York, UK; 3 Liverpool Reviews and Implementation Group, University of Liverpool, Liverpool, UK; 4 Pain Medicine and Neuromodulation, Mid & South Essex University Hospitals, Essex, UK; 5 Leeds Neuromodulation Centre, Leeds Teaching Hospitals, Leeds, UK; 6 Health Economics, QC Medica, Liverpool, UK; 7 Patient and Public Involvement Representatives, Middlesbrough, UK; 8 MRC/CSO Social and Public Health Sciences Unit & Robertson Centre for Biostatistics, Institute of Health and Well Being, University of Glasgow, Glasgow, UK; 9 Saluda Medical Pty Ltd, Artarmon, New South Wales, Australia

We read with interest the letter from Drs Zhou and Sun who raise important points regarding the economic burden of screening trials of spinal cord stimulation (SCS) and how to improve presentation of results.^1^ We appreciate the opportunity to respond and further discuss our study.^2^

We agree on the importance of health economic evaluations of new interventions or newly proposed treatment strategies or approaches. We are very aware of the potential health economic implications of screening trials of SCS. These implications have not been neglected as we have provided a comprehensive assessment of the potential healthcare costs and benefits associated with the use or not of a screening trial prior to implantation of the SCS device. Our health economic work in this area includes a theoretical exercise,^3^ a within-trial cost-utility analysis of the TRIAL-STIM randomised controlled trial,^4^ and a budget impact analysis.^5^ The conclusions were that screening trials of SCS do not represent value for money. In addition, the patient’s perspective has also been considered and showed support for a one-stage procedure without a screening trial.^6^ The rationale for patient preferences for one-stage procedure is that it would save their time, result in only one recovery period and save healthcare resources.^6^

As detailed in the recent TRIAL-STIM report, a cost-utility analysis was not conducted at 36-months as the length of recall required between assessments (i.e., 30-months) would limit interpretation of the results.^2^ We discuss the potential health economic implications in detail in the Supplementary Material 2 and provide herein a summary of the points made. The main point being that in the long-term there were similar rates of adverse events and explants in both groups and there were no statistically or clinically important differences in utility values or pain intensity. Therefore, resource use and associated cost for the follow-up period would be similar in both groups. Given that the main cost differences observed at 6-months were associated with the cost of a screening trial or management of infections which occur at early stages of SCS, we maintain the initial conclusion that screening trials of SCS do not represent value for money.

Zhou and Sun suggest that a plot of the pain intensity score of each patient throughout the duration of treatment “would provide clinicians with the best individual treatment options and assessment of treatment efficacy, when faced with a choice between multiple solutions for the treatment of chronic neuropathic pain”.1 We consider it would be challenging to derive any meaningful interpretation from a plot of n=66 due to overlap of the multiple curves and the long time frame between follow-up assessments. Therefore, we do not believe this would be of value to inform individual treatment options. Our findings are that on average, there is no difference in prognosis with the SCS screening test compared with no screening test. Clinical assessment of the best treatment option for each patient will be based on a number of individual factors and should always be made on a case-by-case basis.

In conclusion, the health economic implications of screening trials of SCS have been considered and thoroughly investigated by the authors. As we state in our paper, we do not propose that screening trials should be eradicated. Instead, an informed decision should be based on professional judgment and patient preferences, considering the advantages and disadvantages of screening trials.^2^
